# Nematicidal Activities of Three Naphthoquinones against the Pine Wood Nematode, *Bursaphelenchus xylophilus*

**DOI:** 10.3390/molecules24203634

**Published:** 2019-10-09

**Authors:** Deok Jea Cha, Junheon Kim, Dong Soo Kim

**Affiliations:** Division of Forest Insects and Diseases Division, National Institute of Forest Science, Seoul 02455, Korea; developcha@korea.kr (D.J.C.); skimds@korea.kr (D.S.K.)

**Keywords:** *Bursaphelenchus xylophilus*, naphthoquinone, reactive oxygen species

## Abstract

*Bursaphelenchus xylophilus* (Steiner & Buhrer) Nickle, is a serious forest pest, causing enormous economic losses in pine trees in Korea, China, Japan, and countries in Western Europe. To prevent pine wilt disease (PWD), trunk injection with nematicide is performed in Korea. Although these nematicidal agents are quite efficient, the development of new nematicidal agents is needed to prevent pesticide resistance and reduce pest management costs. The aim of this study was to investigate nematicidal activities of pure naphthoquinones (NTQs)–1,4-NTQ, juglone, and plumbagin—against *B. xylophilus* via in vitro and semi-in vivo assays to identify new candidate agents for trunk injection. Estimated LC_50_ values (48 h exposure) were 100.0 ppm, 57.0 ppm, and 104.0 ppm for 1,4-NTQ, juglone, and plumbagin, respectively. In the semi-in vivo assay on pine bolt of the Japanese black pine, *Pinus thunbergii*, the population of inoculated *B. xylophilus* was significantly decreased at two weeks after treatment with juglone when compared with the effects of treatment with 1,4-NTQ and plumbagin. We also observed that naphthoquinones could generate reactive oxygen species, which presumably indicated that naphthoquinones caused significant oxidative stress in *B. xylophilus*. The findings of this study suggest the nematicidal potential of naphthoquinones and their possible use in further in vivo assays to test their nematicidal efficacy against *B. xylophilus* when injected through trunk injection.

## 1. Introduction

The pine wilt disease (PWD) caused by the pinewood nematode, *Bursaphelenchus xylophilus* (Steiner & Buhrer) Nickle, results in huge economic losses in coniferous species, especially pines, throughout East Asian countries, including Japan, South Korea, and China [1], as well as in Western Europe [2]. In South Korea, PWD was first reported in Busan in 1988 [3], and after several decades, it eventually spread to most parts of the country. Trunk injection with systemic nematicides is an effective way to manage PWD by directly killing *B. xylophilus,* which is the source of PWD, and is a more environmentally stable strategy of pest control; thus, this strategy is widely used in South Korea [4]. Despite some advantages of trunk injection, improvements in nematicides for trunk injection are required owing to various associated disadvantages, e.g., high cost and possible development of nematicide resistance. Therefore, several studies using plant essential oils and herbal extracts have been conducted to search for novel nematicides that negatively affect the propagation of *B. xylophilus* [5,6,7]. Despite these efforts, no nematicides for trunk injection against *B. xylophilus* have been developed as they have significantly lower efficacy than do conventional nematicides, such as avermectin and emamectin benzoate, for preventing PWD in vivo [8].

Naphthoquinone is a class of organic compounds structurally related to naphthalene and is easily found in nature as products of microorganisms and as secondary metabolites in plants. The bioactivities, such as potential antibacterial, insecticidal, and nematicidal effects, have been studied [9,10,11,12,13,14]. The mechanism of naphthoquinone in organisms is related to enhanced reactive oxygen species (ROS) generation and results in the death of cells [15]. The basic researches on the mechanism of ROS generation by naphthoquinone and its analogs and ROS-related gene expression patterns have been conducted in *Caenorhabditis elegans*. The results of these studies indicated that naphthoquinone caused the generation of ROS, even in low concentrations [16]. In addition, studies on the insecticidal activity of naphthoquinone against the root-lesion nematode *Pratylenchus thornei*, showed its possibility as a nematicide [17].

The objective of this study was to confirm the possibility of the use of naphthoquinone and analogs as a nematicide for trunk injection against PWD by determining the in vitro and semi-in vivo nematicidal activity of pure 1,4-naphthoquinone (1,4-NTQ), 5-hydroxy-1,4-naphthoquinone (juglone), and 5-hydroxy-2-methyl-1,4-naphthoquinone (plumbagin) against *B. xylophilus*. In addition, although several studies revealed that naphthoquinone is a redox cycler that could cause the generation of ROS in various organisms, the nematicidal mechanism of naphthoquinones for *B. xylophilus* has not been elucidated. Thus, we also performed assays on ROS detection to demonstrate that naphthoquinones could be lethal to *B. xylophilus* by inducing oxidative stress.

## 2. Results

### 2.1. In Vitro Nematicidal Activities of Three Naphthoquinones on B. xylophilus

**Figure 1:**
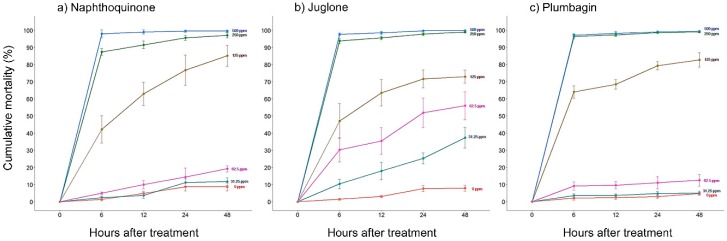
Cumulative mortality (%) in *Bursaphelenchus xylophilus* exposed to serial concentrations from 500 ppm to 31.25 ppm of (**a**) 1,4-naphthoquinone, (**b**) juglone, and (**c**) plumbagin. A 1% dimethyl sulfoxide (DMSO) solution was used as the control. Data are averages of six replicates, and bars represent standard deviations.

The mortality of *B. xylophilus* in the 1% dimethyl sulfoxide (DMSO) control treatment (cumulative mortality = 1–8%) was not significantly different from that observed in the control with water (one-way ANOVA, *F*_1,22_ = 0.095, *p* = 0.761). However, the mortality of *B. xylophilus* was significantly affected by the presence of the three NTQs at concentrations above 62.5 ppm (one-way ANOVA, *p* < 0.05). No significant differences in mortality were found in all treatments with 500 ppm and 250 ppm NTQ solutions and the mortalities at these concentrations were more than 95% at 48 h after treatment (HAT) (Figure 1). Juglone showed the strongest nematicidal activity among the NTQ treatments, although there was no significant difference. LC_50_ values of 1,4-NTQ, juglone, and plumbagin at 48 HAT were 100.0, 57.0, and 103.9 ppm, respectively (Table 1).

### 2.2. Semi-In Vivo Nematicidal Activities of Three Naphthoquinones on B. xylophilus

**Figure 2:**
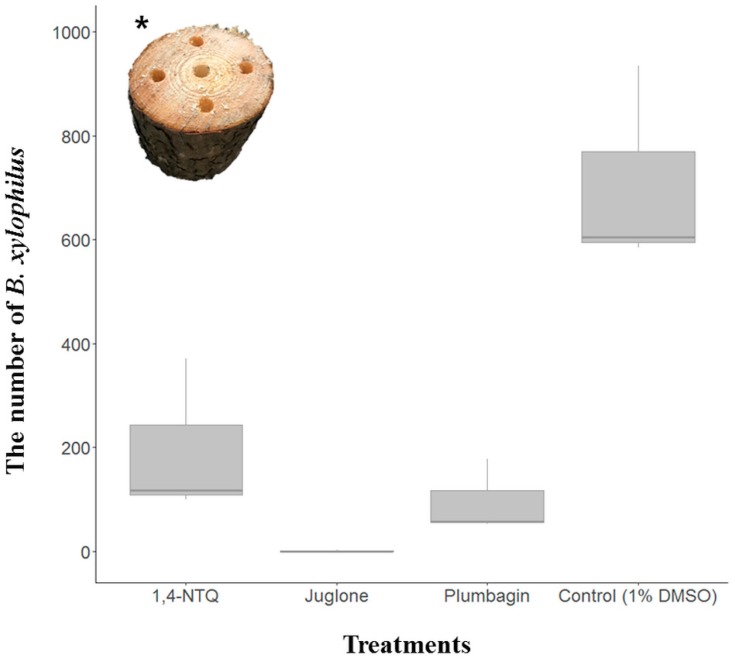
Semi-in vivo assay for nematicidal activities of three naphthoquinones against *Bursaphelenchus xylophilus.* * Picture of the tested pine bloks.

Two weeks after NTQ treatments, the number of *B. xylophilus* in pine blocks were affected by each treatment. In the 1% DMSO treated pine block, the number of *B. xylophilus* per 10 g of pinewood was 708 ± 160. In contrast, the number of *B. xylophilus* was shown to be decreased with all NTQ treatments when compared with the effects of treatment with 1% DMSO. Among NTQ treatments, the number of *B. xylophilus* in the 500 ppm juglone-treated pine block was shown to be significantly decreased (N = 0.67 ± 0.94) when compared with the number of nematodes in 500 ppm 1,4-NTQ- (N = 196 ± 124) and 500 ppm plumbagin-treated (N = 96 ± 58) pine blocks (Figure 2).

### 2.3. Naphthoquinone-Generated ROS Detection

**Figure 3:**
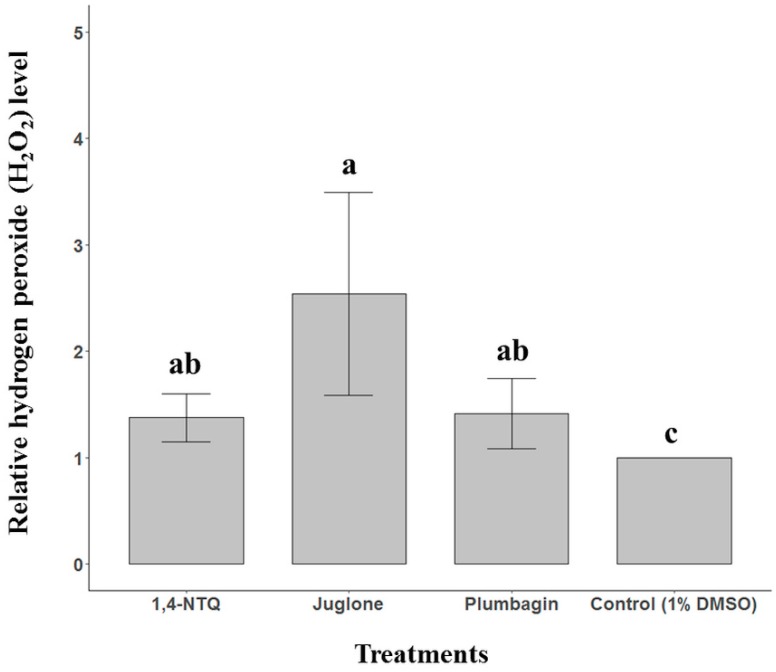
Measurement of H_2_O_2_ levels in *Bursaphelenchus xylophilus* treated with three naphthoquinones in vitro. The differences in letters above the bars represent significantly different statistical values at *p* < 0.05 (Tukey’s test).

**Figure 4:**
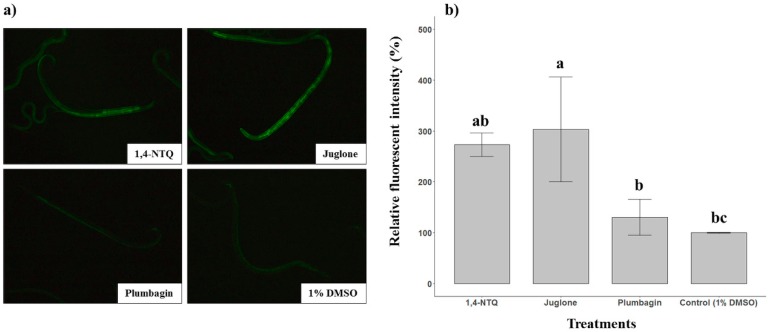
Three naphthoquinones stimulate the reactive oxygen species (ROS) production in *Bursaphelenchus xylophilus* in vitro. (**a**) *B. xylophilus* was incubated with 50 ppm concentrations of 1,4-NTQ, juglone, and plumbagin and a 1% DMSO solution as the control for 3 h, and then treated with 12.5 μM 2′,7′-dichlorofluorescein diacetate for 30 min and photographed by fluorescence microscopy. (**b**) The graph represents relative fluorescent intensity of 2′,7′-dichlorofluorescein generated by the three naphthoquinones compared with that by 1% DMSO. Statistical differences in the means are indicated with different lower-case letters. Bars represent standard deviation.

ROS generation with three naphthoquinones, 1,4-NTQ, juglone, and plumbagin, in *B. xylophilus,* was evaluated using two methods. First, the Amplex red hydrogen peroxide assay kit, 10-acetyl-3,7-dihydroxyphenoxazine, was used to selectively detect H_2_O_2_ [18]. Second, using the 2′,7′-dichlorofluorescein diacetate (DCFDA) assay, fluorescent dye staining was used to detect all induced ROS [19]. In the result of the Amplex red hydrogen peroxide assay, treatment of *B. xylophilus* with 50 ppm juglone generated 1.5–2.5 times more H_2_O_2_ than the 1% DMSO treatment, taken as the control (*p* < 0.05). However, treatment of *B. xylophilus* with 50 ppm 1,4-NTQ or plumbagin did not show a difference in the H_2_O_2_ level when compared with the effects of the 1% DMSO treatment (Figure 3). Next, in the result of the DCFDA assay, among all treatments, treatment of *B. xylophilus* with 50 ppm juglone and 1,4-NTQ showed higher fluorescence intensity than did the 1% DMSO treatment (Figure 4).

## 3. Discussion

Through the in vitro and semi-in vivo experiments, it was revealed that the three NTQs had nematicidal activity within short periods following exposure. NTQs have been reported for their insecticidal and nematicidal activity [11,12,17,20,21]. Among the NTQ analogs, juglone and plumbagin showed the strongest acaricidal activities against *P. cuniculi* [14]. Plumbagin obtained from persimmon root, *Diospyros kaki,* had the most potent larvicidal activity against *Aedes aegypti*, and *Ochlerotatus togoi* [22].

**Figure 5:**
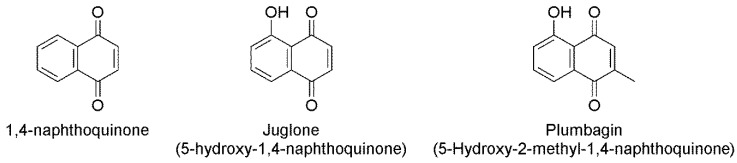
Structure of the three naphthoquinones.

The potential insecticidal activity of 1,4-naphthoquinone structural derivatives has been established [22,23]. Juglone and plumbagin have a hydroxyl group at the 5- position, and plumbagin has a methyl group at the 2- position in addition to the naphthoquinone structure (Figure 5). The type and placement of functional groups on the skeleton of cyclic compounds determine their biological efficacy [12]. For example, phenolic monoterpenoids, e.g., thymol and carvacrol, showed higher nematicidal activity against *B. xylophilus* among all monoterpenoids [24]. In aliphatic compounds, alcohols exhibited higher nematicidal activity than did hydrocarbons, which have the same skeleton [25]. In our results, juglone showed the highest nematicidal activity, consistent with the findings of several studies on the nematicidal activity of naphthoquinone wherein juglone showed the highest nematicidal activity when compared with other naphthoquinone analogs [11,17].

We performed a semi-in vivo experiment using pine blocks. To the best of our knowledge, this is the first example of a bioassay conducted for testing nematicidal activity against *B. xylophilus*. Results similar to those of the in vitro test were obtained. Results of the semi-in vivo test suggested that after being absorbed into the pine blocks, the three naphthoquinones effectively inhibited the population growth of *B. xylophilus*. Therefore, this bioassay method would be a useful test for nematicidal activity. Unlike the results in pine blocks, nematicidal activities of naphthoquinones against *B. xylophilus* might be different in living pine trees. However, it is obvious that the number of *B. xylophilus* did not significantly increase even after two weeks in the dark condition after treatment with naphthoquinones. Thus, the results of our semi-in vivo experiment show the possibility of applying naphthoquinones through trunk injection to pine trees to prevent infection of *B. xylophilus*.

ROS was measured to understand the mechanism of NTQs. The results of the Amplex red hydrogen peroxide assay revealed that the treatment of *B. xylophilus* with NTQ has a large effect on intracellular ROS production in *B. xylophilus*. However, no difference was observed in fluorescence intensity in the case of the treatment of *B. xylophilus* with 50 ppm plumbagin compared to the 1% DMSO treatment. This result is probably because the treatment concentration (50 ppm) of plumbagin in the bioassay was lower than the LC_50_ concentration of plumbagin. However, in the case of 1,4-NTQ, the fluorescence level at the same concentration was higher than that of plumbagin. Although both naphthoquinone analogs had a similar range of LC_50_ concentration in our in vitro result, in low concentration, among the 62.5 ppm to 31.25 ppm treatments, 1,4-NTQ showed slightly higher mortality than plumbagin (Figure 1). Therefore, the difference in fluorescence intensity between 1,4-NTQ and plumbagin would have been caused by their different nematicidal activities on *B. xylophilus* at the low concentration. Several studies revealed that quinones stimulate the production of ROS in various organisms [9,10,11,12,13,14]. Our results from the two assays also showed that naphthoquinone could obviously affect the generation of ROS in *B. xylophilus,* and thus, it was speculated that the nematicidal activity on *B. xylophilus* was caused by the ROS produced by the naphthoquinone.

## 4. Materials and Methods

### 4.1. Nematodes

*B. xylophilus* was provided by the National Institute of Forest Science, Seoul, Korea. The identification of *B. xylophilus* was confirmed using morphological characters and genetic differences, which were confirmed by RFLP method [26]. *B. xylophilus* were reared on a fungal mat of *Botrytis cinerea* on potato dextrose agar media at 25 ± 1 °C and 40% humidity for several generations.

### 4.2. Nematicidal Activities of Three Naphthoquinones on B. xylophilus In Vitro

Three naphthoquinones (NTQs), 1,4-NTQ, juglone, and plumbagin (Sigma-Aldrich, St. Louis, MO, USA), were solubilized in 1% DMSO solution (laboratory grade) to obtain the final NTQ concentrations of 500, 250, 125, 62.5, and 31.25 ppm using half-fold serial dilutions, and a 1% DMSO solution was used as the control (mortality with the 1% DMSO solution was not different from that in the untreated groups using water). Working solutions of NTQs were prepared and used on the day of the experiment. Each treatment comprised six replicates, and each mortality experiment was repeated thrice. Approximately 300 nematodes were placed on each well of a 6-well cell culture plate containing 3 mL of each prepared NTQ solution and 1% DMSO solution (control). Then, the 6-well cell culture plates were maintained at 25 ± 1 °C and 40% humidity, in the dark, and nematode mortality was observed at 6, 12, 24, and 48 h after treatment (HAT). Nematodes were considered as dead if their bodies were straight and when they did not move, even after transferring to clean water.

### 4.3. Semi-In Vivo Bioassay

The pine blocks (*P. thunbergii*, 10 cm width and 15 cm long) were sterilized using an autoclave (121 °C for 30 min). Then, 30 mL of 500 ppm of each NTQ (1,4-NTQ, juglone, and plumbagin) solution and 1% DMSO solution as the control were absorbed through the hall by drilling from the pine blocks under dark conditions. After complete absorption, approximately 4500 individuals of *B. xylophilus* per 1 mL were inoculated in each pine block. Finally, the treated pine blocks were incubated at 25 ± 1 °C and 40% humidity for 2 weeks in the dark. Two weeks after treatment, 10 g of pinewood chips was obtained from the upper, middle, and bottom parts of the block by drilling, the nematodes were extracted from the chips of pinewood using the Baermann funnel method, and the number of *B. xylophilus* were counted under a microscope (Leica, Wetzlar, Germany).

### 4.4. Naphthoquinone-Generated ROS Detection

The measurement of ROS produced by naphthoquinones in *B. xylophilus* was performed using two methods. First, H_2_O_2_ produced as a result of ROS production was measured using the Amplex red hydrogen peroxide assay kit (Invitrogen, Seoul, Korea) according to the manufacturer′s instructions with some modification. Approximately 300 male adult individuals of *B. xylophilus* were collected and washed thrice with ddH_2_O to eliminate *Botrytis cinerea*. *B. xylophilus* individuals were transferred to a 1.5 mL tube containing 50 ppm working solution of each NTQ and 1% DMSO solution as the control. Then, the tubes were incubated at 25 ± 1 °C for 3 h in the dark. After incubation, each treatment was washed thrice with 1X Amplex reaction buffer. Fifty microliters of the buffer with approximately 300 live *B. xylophilus* individuals or standard H_2_O_2_ solutions (2.5 μM to 40 μM) was mixed with 50 μL of Amplex Red working solution (100 μM Amplex^®^ Red reagent with 0.2 U/mL HRP, Waltham, MA, USA) at each well in a 96-well plate and incubated for 3 h at 25 ± 1 °C in the dark. After incubation, H_2_O_2_ was measured with a microplate reader (EPOCH2, BioTek, Winooski, VT, USA) at 570 nm. Data of absorbance levels from H_2_O_2_ produced by NTQ treatments was calculated in comparison to the relative values of the control (1% DMSO solution). Second, the levels of ROS were measured using 2′,7′-dichlorofluorescein-diacetate (DCFDA), which is activated by ROS. Approximately 300 adult individuals of *B. xylophilus* were collected and washed thrice with ddH_2_O to eliminate *Botrytis cinerea*. *B. xylophilus* were transferred to a 1.5 mL tube containing 50 ppm of each NTQ working solution and 1% DMSO solution as the control. Then, the tubes were incubated at 25 ± 1 °C for 3 h in the dark. After incubation, each treatment was washed thrice with ddH_2_O. The ddH_2_O (500 μL) with live *B. xylophilus* was mixed with 500 μL of 25 μM DCFDA solution (final volume 12.5 μM) and incubated at 25 ± 1 °C for 30 min in the dark. After incubation, each treatment was washed thrice with ddH_2_O to eliminate the unreacted DCFDA in the solution. Then, *B. xylophilus* were mounted on a glass slide for examination with a fluorescence microscope system (Leica DM6 with CTR 6 LED, Wetzlar, Germany) at 488 nm of excitation wavelength and 510 nm of emission filter. Finally, fluorescence intensities were semi-quantified as relative fluorescent units measured by ImageJ (ver. 1.8.0, https://imagej.nih.gov/ij/index.html). For detecting ROS, we only used adult males of *B. xylophilus* as the female varies in body size depending on ovulation status. In addition, we limited the treatment time to 3 h to keep *B. xylophilus* alive in order to exclude ROS values measured from dead *B. xylophilus* affected by NTQ or natural longevity.

### 4.5. Statistics

The mortality of each NTQ treatment at each concentration was transformed to arcsine square-root and compared using one-way ANOVA. The LC values were estimated by probit analysis with dose-response data at 48 HAT [27]. Statistical analysis was performed using R (ver. 3.5.2, https://cran.r-project.org/mirrors.html) [28]. Untransformed data are shown.

## 5. Conclusions

In this study, three NTQs–1,4-NTQ, juglone, and plumbagin were investigated for their nematicidal activities against *B. xylophilus* for the first time. In addition, this may be the first report of the semi-in vivo bioassay method. Our results indicate that NTQs could be alternative nematicidal agents applied via trunk injection. However, further studies, including their formulation and application on living trees, are necessary for practical use.

## Figures and Tables

**Table 1:** The median lethal concentration (LC_50_) values of naphthoquinones against *Bursaphelenchus xylophilus* mixed development stages in vitro at 48 h after exposure to 1,4-naphthoquinone, juglone, and plumbagin.

Compounds	LC_50_ (ppm) ^1^	95% CI (ppm) ^2^	*R* Square	Regression Line
1,4-NTQ (1,4-Naphthoquinone)	100	60–140	*R*^2^ = 0.8489	y = 92.086x − 134.17
Juglone (5-Hydroxy-1,4-naphthoquinone)	56.98	16.98–96.98	*R*^2^ = 0.9238	y = 60.502x − 56.227
Plumbagin (5-Hydroxy-2-methyl-1,4-naphthoquinone)	103.86	63.86–143.86	*R*^2^ = 0.8512	y = 95.707x − 142.99

^1^ LC_50_ was analyzed according to the mortality (%) of nematodes at 48 h. ^2^ CI: confidence interval.
